# Effect of Intravitreal Methotrexate and Rituximab on Interleukin-10 Levels in Aqueous Humor of Treated Eyes with Vitreoretinal Lymphoma

**DOI:** 10.1371/journal.pone.0065627

**Published:** 2013-06-04

**Authors:** Harish Raja, Melissa R. Snyder, Patrick B. Johnston, Brian P. O’Neill, Juline N. Caraballo, Joseph G. Balsanek, Brian E. Peters, Paul A. Decker, Jose S. Pulido

**Affiliations:** 1 Mayo School of Graduate Medical Education, Mayo Clinic, Rochester, Minnesota, United States of America; 2 Department of Laboratory Medicine and Pathology, Antibody Immunology Laboratory, Mayo Clinic, Rochester, Minnesota, United States of America; 3 Department of Hematology, Mayo Clinic, Rochester, Minnesota, United States of America; 4 Department of Neurology, Mayo Clinic, Rochester, Minnesota, United States of America; 5 Department of Ophthalmology, Mayo Clinic, Rochester, Minnesota, United States of America; 6 Department of Immunology, Mayo Clinic, Rochester, Minnesota, United States of America; 7 Department of Biomedical Statistics, Mayo Clinic, Rochester, Minnesota, United States of America; National Eye Institute, United States of America

## Abstract

Intraocular cytokines are promising diagnostic biomarkers of vitreoretinal lymphoma. Here, we evaluate the utility of IL-10, IL-6 and IL-10/IL-6 for discriminating lymphoma from uveitis and report the effects of intraocular methotrexate and rituximab on aqueous cytokine levels in eyes with lymphoma. This is a retrospective case series including 10 patients with lymphoma and 7 patients with uveitis. Non-parametric Mann-Whitney analysis was performed to determine statistical significance of difference in interleukin levels between lymphoma and uveitis. Compared to eyes with uveitis, eyes with lymphoma had higher levels of IL-10 (U = 7.0; two-tailed p = 0.004) and IL-10/IL-6 (U = 6.0; two-tailed p = 0.003), whereas IL-6 levels were more elevated, although insignificant, in those patients with uveitis than in lymphoma (U = 15.0; two-tailed p = ns). Using a receiver operating characteristic analysis to identify threshold values diagnostic for lymphoma, optimal sensitivity and specificity improved to 80.0% and 100%, respectively, for IL-10>7.025 pg/ml and 90.0% and 100.0%, respectively, for IL-10/IL-6>0.02718. In patients in whom serial interleukin levels were available, regular intravitreal treatment with methotrexate and rituximab was associated with reduction in IL-10 levels over time. In conclusion, optimal IL-10 and IL-10/IL-6 threshold values are associated with a diagnostic sensitivity ≥80% and specificity of 100%. Therefore, these cytokines may serve as a useful adjunct in the diagnosis of lymphoma. While negative IL-10 and IL-10/IL-6 values do not exclude a diagnosis of lymphoma, elevated levels do appear to be consistent with lymphoma clinically. Moreover, elevated levels of IL-10 in the setting of a clinically quiet eye may point to impending disease recurrence. Lastly, once lymphoma is diagnosed, IL-10 levels can be monitored over time to assess disease activity and therapeutic response.

## Introduction

There are three classes of intraocular lymphoma. Primary vitreoretinal lymphoma (PVRL), a subset of primary central nervous system lymphoma (PCNSL), may present with or without CNS disease at the time of ocular diagnosis. Secondary intraocular lymphoma occurs due to metastatic spread of systemic lymphoma. A third, and entirely distinct entity, is primary lymphoma of the choroid. [1]

Lymphoma often presents with nonspecific symptoms such as blurred vision and floaters. [2], [3] On slit lamp examination, cell and flare in the anterior chamber and infiltrate in the vitreous and sub-retinal pigment epithelial space are typical findings. [2] Patients may even transiently improve after being treated with topical corticosteroids when the diagnosis is thought to be uveitis, further confounding the diagnosis. [4] For these reasons, many patients with lymphoma undergo a protracted workup before a correct diagnosis is established. [3], [5]

There is currently no optimal diagnostic test for lymphoma, although the gold standard remains pars plana vitrectomy (PPV) with cytologic analysis of the vitreous or retina. While highly sensitive and specific under optimal conditions, cytology is associated with a significant false-negative rate. [6] Considerable effort has, thus, focused on identifying noninvasive tools to aid in the diagnosis of lymphoma. These include molecular analysis of gene rearrangements, immunohistochemistry/flow cytometry analysis of cell surface markers, and quantification of intraocular cytokines. Interleukin (IL)-10, a cytokine of interest, is selectively expressed in malignancy and has been shown to function in stimulating B-cell antibody production [7] and evading cellular immunity. [8] Alternatively, IL-6 is elevated in the setting of inflammation unrelated to malignancy. [9]

Given the expression profiles of these cytokines, prior studies have sought to identify optimal threshold values of IL-10 [5], [9], [10], [11] and the ratio of IL-10 to IL-6 [10] as indicators of malignancy. Levels of IL-10 in the vitreous have been shown to be elevated in patients with intraocular lymphoma [5] and significantly higher in eyes with lymphoma compared to uveitis. [10] In one study, threshold IL-10>100 pg/mL and IL-10/IL-6>1.0 correctly identified 18/22 (82%) patients with lymphoma. [10] In apparent concordance with these findings, one study found aqueous IL-10 levels to be similarly higher in lymphoma compared to uveitis. Furthermore, in this same study, diagnostic accuracy was comparable whether or not utilizing aqueous or vitreous threshold values was done; aqueous IL-10 level >50 pg/mL was associated with a sensitivity and specificity of 0.89 and 0.93, respectively, compared to 0.99 and 0.89, respectively, for vitreous IL-10 level >400 pg/mL. [11].

## Materials and Methods

This study was approved by the Mayo Clinic Institutional Review Board, Rochester, MN. Informed consent was not required from human participants since the data were analyzed anonymously. All clinical investigation was conducted according to the principles expressed in the Declaration of Helsinki.

The Institutional Review Board waived the need for written informed consent from the participants. The above-referenced application is approved by expedited review procedures (45 CFR 46.110, item 5). The Reviewer conducted a risk-benefit analysis and determined the study constitutes minimal risk research. The Reviewer determined that this research satisfies the requirements of 45 CFR 46.111. The Reviewer approved waiver of the requirement to obtain informed consent in accordance with 45 CFR 46.116 as justified by the Investigator and waiver of HIPAA authorization in accordance with applicable HIPAA regulations.

All patients who were evaluated for possible lymphoma within an 18-month time frame were included in this study. This included those patients who were ultimately diagnosed with lymphoma and those who ended up with a diagnosis of uveitis but had suspicious findings that required vitrectomy in five of seven cases. Of those patients diagnosed with uveitis, none have developed evidence of vitreoretinal or CNS lymphoma to date. Each patient had an anterior chamber paracentesis performed one or more times throughout the course of being seen at our clinic. Each time, 0.1 cc of aqueous humor was removed. Interleukin levels were measured in undiluted aqueous samples using a bead-based assay run on a Luminex-based platform (manufacturer: Affymetrix). Linearity limit of detection was used to establish the reportable range, and inter- and intra- assay precision were verified. The same kit lot and provider were used in all determinations. A review of medical records was completed; data on IL-10 and IL-6 levels as well as patients’ disease and treatment course were recorded.

Statistical analysis was performed using GraphPad Prism. [12] In our analysis, we aimed to identify optimal sensitivities and specificities threshold values associated with each of the three markers of interest. For each patient with lymphoma, the highest IL-10 and lowest IL-6 values were chosen. The converse was done when selecting IL-10 and IL-6 values for each patient with uveitis. The ratio of IL-10 to IL-6 was then calculated for each patient. A receiver operating characteristic (ROC) analysis was performed to identify optimal threshold values of the IL-10, IL-6, and IL-10/IL-6 markers for discriminating lymphoma from uveitis. Additionally, the distribution of cytokine values between lymphoma and uveitis were compared using a non-parametric Mann-Whitney-Wilcoxon analysis.

## Results

A review of medical records was completed, and aqueous levels of IL-10 and IL-6 from 10 patients with diffuse large B cell vitreoretinal lymphoma and 7 patients with uveitis are reported. See Table 1 for lymphoma case histories, Table 2 for dates of intraocular treatment and IL levels in patients with lymphoma, and Table 3 for uveitis case histories and IL levels.

**Table 1:** Profiles of patients with vitreoretinal lymphoma (Cases 1–10).

Case #	Age/sex	Comorbidities	VA _Initial_	VA _Recent_	Date of initial diagnosis	Site of initial diagnosis	Histologic subtype of lymphoma & Staging	Primary treatment	Response to primary treatment	Date of ocular diagnosis	Ocular treatment	Extra - ocular disease at time of ocular diagnosis?	CNS involvement?	Dates and sites of relapse	Treatment for relapse	Patient expired?
Case 1	66-year-old Caucasian female	Type II diabetes mellitus	OD: 20/60^+1^	OD: 20/25^−2^	07/07/2009	Brain (multifocal)	Diffuse large B-cell non-Hodgkin lymphoma	Systemic MTX and RTX (07/2009–10/2009)	CR	01/20/2010 OS: vitreous pathology from PPV	Intraocular MTX, RTX and dexamethasone	No	Yes	Ocular relapse (09/20/10) OS: vitreous cytology from PPV	Continued intraocular RTX and dexamethasone	No
			OS: 20/40^−2^	OS: 20/30						02/03/2010 OD: vitreous cytology from PPV				CNS relapse (05/01/2011)	Systemic temozolomide and RTX; tumor growth progressed, and patient was started on MTX	
Case 2	53- year-old male	Hypertension	OD: 20/50 (NI)	OD:–	09/19/2007	Ocular	Diffuse large B-cell non-Hodgkin lymphoma	High dose systemic MTX; the patient was not a good candidate for intraocular treatment and instead received 34 Gy of radiation OU (04/07/2008-04/29/2008)	CR	Same as initial diagnosis	Same as primary treatment	No	No	Systemic relapse with skin and nodal involvement (07/2009)	Induction with three cycles of R-CHOP^a^, followed by consolidation with BEAM^b^ conditioning and autologous SCT (10/26/2009) to CR	No
			OS: 20/30^−1^ (NI)	OS: 20/200^−1^		OD: retinal/choroidal pathology from PPV				Pathology proven disease OD with clinically diagnosed disease OS						
Case 3	73-year-old Caucasian Male	Chronic renal insufficiency	OD: 20/25	OD: 20/150^+1^ (20/100)	05/01/2009	Systemic chronic lymphocytic leukemia	Richter’s transformation of CLL (Diffuse large B cell lymphoma)	None	N/A	04/09/2010 OS: Vitreous and retinal pathology from PPV consistent with Richter’s transformation	Intraocular and high dose systemic MTX; the patient responded well but developed renal failure, likely from MTX, and was switched to temozolomide and RTX (06/2010). There was progression of disease, and the patient underwent 36 Gy of WBRT including the eye fields to remission (09/2010)	Yes	Yes (parietal lobe lesion)	None	N/A	Yes (05/2011) From unknown cause
			OS: HM	OS: 200E at 2′						Pathology proven disease OS and clinically diagnosed disease OD						
Case 4	80-year-old Caucasian male	None	OD: 20/20^−2^	OD: 20/20^−1^	12/05/1996	Left testicle	Diffuse large B-cell non-Hodgkin lymphoma	Six cycles of CHOP^c^ chemotherapy and intrathecal MTX,+radiation to the right testicle	CR	07/26/2010 OD: vitreous pathology from PPV (relapse after testicular tumor)	Six cycles of high dose MTX to PR; there was disease progression within two months, and the patient was treated with gamma knife to the right parietal lesion (12/22/2010) and 40 Gy of WBRT (04/11/11–05/06/11 to CR	Yes (cerebrum); no systemic disease	Yes	None	N/A	No
			OS: 20/20^−1^	OS: 20/25^−2^ (NI)						Unilateral, pathology proven disease OD						
Case 5	61-year-old Caucasian female	Hypertension, history of deep vein thrombosis	OD: 20/25^−22^	OD: 20/25	10/26/2010	CNS (multifocal); also found to have ocular disease at the time of diagnosis by ophthalmic exam	Diffuse large B-cell Lymphoma	High dose systemic MTX with plan to proceed to SCT transplant; the patient was not in CR when transplant was performed, but lymphoma was determined to be chemotherapy- sensitive	N/A	02/21/11 OS: vitreous pathology from PPV	Intraocular MTX and RTX; the patient underwent consolidation with BEAM^b^ conditioning and autologous SCT (03/07/2011)	Yes	Yes	CNS (CSF) (06/11/2011)	Craniospinal irradiation followed by WBRT (06/27/11–07/11/11) to CR	No
			OS: 20/20^−^	OS: 20/150^+2^ (20/80^−1^)						Pathology proven disease OS and clinically diagnosed disease OD						
Case 6	51-year- old Caucasian female	Sarcoidosis	OD: 20/70 (NI)	OD: 20/30^−1^ (NI) (NI)	04/23/2007	CNS lesion (unclear whether CNS or ocular disease developed first); the patient developed ocular symptoms prior to diagnosis of CNS disease, and was diagnosed with uveitis at an outside provider.	Diffuse large B-cell lymphoma	Initially received corticosteroids for presumed uveitis; the patient then received high dose MTX to CR, followed by consolidation with BEAM^b^ conditioning and autologous SCT (10/04/2007)	CR	08/01/2007 clinically diagnosed bilateral disease	Intraocular bevacizumab	Yes	Yes	CNS (08/19/2008)	Reinitiated high dose MTX	Yes (05/2011) due to disease progression
			OS: 20/70 (NI)	OS: 20/40^+1^												
Case 7	69-year-old Caucasian male	Spindle table-1-captioncell lung table-1-captioncancer diagnosed 06/2009, hypothyroidism, history of seizures	OD: 20/40^−2^ (NI)	OD: 20/20	02/10/1999	Nasal septal mass	Diffuse large B-cell lymphoma	CHOP^c^ chemotherapy and prophylactic brain irradiation	CR	01/20/2010 OS: vitreous cytology and pathology from PPV	Intraocular MTX and RTX in combination with systemic temozolomide, high dose MTX, and RTX	Yes (CSF)	Yes	Testicular	Orchiectomy and maintenance RTX	Yes (12/2010)
			OS: 20/50-2 (NI)	OS: 20/60^−2^						Cytology and pathology proven disease OS and clinically diagnosed disease OD				Neurolymphomatosis (11/05/2008)	High dose MTX until 01/2010	
														Pelvis (low grade) 05/2010	Systemic, bendamustine, high dose MTX, and RTX+radiation	
Case 8	54-year-old Caucasian male	None	OD: 20/20^−2^ (PH)	OD: 20/60^−2^ (20/40^−2^)	10/01/2008	Systemic (bone marrow negative)	Diffuse large B-cell lymphoma	Induction with high dose MTX and six cycles of R-CHOP^c^, followed by consolidation with BEAM^b^ conditioning and autologous SCT (03/30/2009)	CR	10/01/2008 OD: vitreous cytology from PPV	Variable intraocular RTX	No	No	Ocular relapse (10/11/2010)	Intraocular MTX and RTX	No
			OS: 20/20 (PH)	OS: 20/25^−2^												
Case 9	68- year-old Caucasian female	Breast cancer, hypertension	OD: 20/70^+1^ (NI)	OD: 20/80 (NI)	07/31/2009	CNS	Diffuse large B-cell lymphoma	Induction with high dose systemic MTX to CR, followed by consolidation with BEAM^b^ conditioning and autologous SCT (01/28/10); the patient was seen in 12/2009 by an outside provider for ocular symptoms and treated with corticosteroids for presumed optic neuritis	CR	01/2010 Clinically diagnosed disease OU despite negative paracentesis	Intraocular MTX and RTX starting from 01/2010	Yes	Yes (CNS)	None	N/A	No
			OS: 20/60^+1^ (NI)	OS: 20/25^−2^						02/11/2011 OD: vitreous pathology from PPV						
										Pathology proven disease OD and clinically diagnosed disease OS						
Case 10	72-year-old Caucasian female	None	OD: 20/40^+1^ (20/25^−2^)	OD: 20/50^−2^ (20/40^−2^)	09/27/2010 OU: PPV outside provider positive for diffuse large B cell lymphoma	Ocular	Diffuse large B-cell lymphoma	No evidence of intraocular treatment prior to her first visit to our clinic in 12/2010; the patient underwent PPV with intraoperative administration of MTX and RTX (05/2011); also received intraocular MTX and RTX in both eyes at outside provider (09/2011)	N/A	Same as initial diagnosis	Same as initial treatment	No	Yes	CNS (05/2011)	Six cycles of systemic temozolomide, MTX, and RTX to CR of CNS lymphoma	No
			OS: 20/25^−2^ (NI)	OS: 20/30^+2^ (20/25^−1^)						09/2011 PPV at outside provider				Bilateral ocular disease (12/2011)	Outside provider did not repeat intraocular injections of MTX/RTX due to intraocular pressure spike	

*CLL Chronic lymphocytic lymphoma, CNS* Central nervous system, *CR* Complete remission, *MTX* Methotrexate, *N/A* Not applicable, *NI* No improvement, *OD* Oculus dexter, *OS* Oculus sinister, *OU* oculus uterque, *PH* Pinhole, *PR* Partial remission, *PPV* Pars plana vitrectomy, *RTX* Rituximab *SCT* Stem cell transplant, *WBRT* Whole brain radiotherapy *VA* Visual acuity.

a R-CHOP: Rituximab, Cyclophosphamide, Hydroxydaunorubicin, Oncovin, Prednisone.

b BEAM: Bendamustine, Cytarabine, Etoposide and Melphalan.

c CHOP: Cyclophosphamide, Hydroxydaunorubicin, Oncovin, Prednisone.

**Table 2:** Interleukin concentrations and dates of intraocular treatment in patients with vitreoretinal lymphoma (Cases 1–10).

Case #	Interleukin-10 concentration (pg/mL)	Interleukin-6 concentration (pg/mL)	Treatment dates/treatment regimens	Eye exam findings
Case 1	09/08/2010: OD: –;OS: 152.41	09/08/2010:OD: –;OS: 6.9		09/08/2010: OS: A/C: Deep and quiet; Vitreous: “Some cells trapped between posterior capsule and residual hyaloids face; no cells deeper in vitreous”
			01/20/2010:OS: Dexamethasone 400 mcg/0.1 cc	
			02/02/2010: OS: Dexamethasone 400 mcg/0.1 cc; RTX 1 mg/0.1 cc	
			02/03/2010: OD: Dexamethasone 320 mcg/0.8 cc; RTX 1 mg/0.1 cc	
			05/14/2010: OS: MTX 400 mcg/0.1 cc	
			09/08/2010: OS: Dexamethasone 400 mcg/0.1 cc; RTX 1 mg/0.1 cc	
Case 2	02/17/2011: OS: 0.56	02/17/2011: OS: 211.79		02/17/2011: OS: A/C: –; Vitreous: 1+ cell
			08/15/2007: OD: Dexamethasone	
			10/17/2007: OD: Dexamethasone 400 mcg	
			12/15/2007: OD: Dexamethasone 400 mcg; MTX 400 mcg	
Case 3			05/20/2010: OD: MTX 400 mcg/0.1cc	
	07/09/2010: OD: 2.04; OS: 72.19	07/09/2010: OD: 1.67; OS: 124.21	07/09/2010: OU: MTX 400 mcg/0.1 cc	07/09/2010: OD: A/C: 2+ flare; Vitreous: 2+ cell, dense syneresis, Weiss ring;OS: A/C: 4+ flare; Vitreous: 2+ cell
Case 4	08/26/2010: OD: 33.74	08/26/2010: OD: 278.75	08/26/2010: OD: MTX 400 mcg/0.1 cc; RTX 1 mg/0.1 cc	08/26/2010: No eye exam available
Case 5				02/14/2011:OD: A/C: Deep and quiet; Vitreous: Trace cells; OS: A/C: Deep and quiet; Vitreous: 1+ cell
	02/21/2011: OD:–; OS: 0.70	02/21/2011: OD:–; OS: 1.67	02/21/2011: OS: MTX 400 mcg/0.1 cc; RTX 1 mg/0.1 cc	
	03/28/2011: OD: 1.73; OS: 0.56	03/28/2011: OD: 20.84; OS: 9.77	03/28/2011: OU: MTX 400 mcg/0.1 cc; RTX 1 mg/0.1 cc	03/28/2011: Injection only; No eye exam available
	05/05/2011: OD: 1.72; OS: 0.56	05/05/2011: OD: 12.21; OS: 8.63	05/5/2011: OU: MTX 400 mcg/0.1 cc; RTX 1 mg/0.1 cc	05/05/2011: OD: A/C: Cataract; Vitreous: Some cells pigmented;OS: A/C: 0.5–1+ cell; Vitreous: 0.5–1+ cell
Case 6			02/2007: OD: Corticosteroids	
			08/06/2007: OS: RTX 1.25 mg	
	02/04/2011: OD: 9.59; OS:–	02/04/2011: OD: 356.28; OS:–		02/04/2011: OD: A/C: No cells; Vitreous: Trace cells; OS: A/C: No cells; Vitreous: No cells
	03/24/2011: OD: –; OS: 0.56	03/24/2011: OD: –; OS: 277.38		03/24/2011: No eye exam available
Case 7			01/11/2010: OD: MTX 400 mcg/0.1 cc; RTX 1 mg/0.1 cc	
			01/19/2010: OD: MTX 400 mcg/0.1 cc; RTX 1 mg/0.1 cc	
			04/8/2010: OS: MTX 400 mcg/0.1 cc; RTX 1 mg/0.1 cc	
			04/22/2010: OD: MTX 400 mcg/0.1 cc; RTX 1 mg/0.1cc	
	07/06/2010: OD: 10.77; OS: 34.45	07/06/2010: OD: 359.07; OS: 960.45	07/06/2010: OU: MTX 400 mcg/0.1 cc; RTX 1 mg/0.1 cc	07/06/2010: OD: A/C: Deep and quiet; Vitreous: 2 to 3+ cells; OS: A/C: Deep and quiet; Vitreous: 2+ cells
	08/16/2010: OD: 1.11; OS: 0.56	08/16/2010: OD: 85.85; OS: 165.03	08/16/2010: OU: RTX 1 mg/0.1 cc	08/16/2010: OD: A/C: Deep and quiet; Vitreous: 2+ cells (better than before); OS: A/C: Deep and quiet; Vitreous: 1+ cells
	11/03/2010: OD: 3.68; OS: 12.81	11/03/2010: OD: 46.03; OS: 96.73	11/3/2010: OU: MTX 400 mcg/0.1 cc; RTX 1 mg/0.1 cc	11/03/2010: OD: A/C: Deep and quiet; Vitreous: 2+ cells (not improving);OS: A/C: Deep and quiet; Vitreous: 2+ cells
Case 8			01/01/2009: OU: RTX 1 mg/0.1 cc	
			01/26/2009: OU: Ttriamcinolone 4 mg	
			02/03/2009: OU: RTX 1 mg/0.1 cc	
	10/11/2010: OD: 9.21; OS:–	10/11/2010: OD: 10.58; OS:–	10/11/2010: OD: Dexamethasone 400 mcg/0.1cc; MTX 400 mcg/0.1 cc; RTX 1 mg/0.1 cc	10/11/2010: No eye exam available
	10/28/2010: OD: 6.54; OS: 0.56	10/28/2010: OD: 33.19; OS: 2.93	10/28/2010: OU: Dexamethasone 400 mcg/0.1 cc; MTX 400 mcg/0.1 cc; RTX 1 mg/0.1 cc	10/28/2010 : Injection only; No eye exam available
			11/11/2010: OU MTX 400 mcg/0.1 cc	
			11/18/2010: OU: RTX 1 mg/0.1 cc	
			11/29/2010: OU: MTX 400 mcg/0.1 cc	
	12/20/2010: OD: 0.56; OS: 0.56	12/20/2010: OD: 388.67; OS: 1.67	12/20/2010: OU: RTX 1 mg/0.1 cc	12/20/2010: Injection only; No eye exam available
	01/26/2011: OD: 2.12; OS: 0.56	01/26/2011: OD: 9.79; OS: 1.94	1/26/2011: OU: MTX 400 mcg/0.1 cc	1/26/2011: OD: A/C : Rare cells; Vitreous: 1+ cells in the mid vitreous, skirt seen; OS: A/C Deep and quiet; Vitreous: Trace pigmented and white cells, post vitreous detachment
			02/22/2011: OU: MTX 400 mcg/0.1 cc; RTX 1 mg/0.1 cc	
	03/16/2011: OD: 0.56; OS: 0.56	03/16/2011: OD: 11.22; OS: 2.22	03/16/2011: OU : MTX 400; mcg/0.1 cc; RTX 1 mg/0.1 cc	03/16/2011: OD: A/C: Rare cells; Vitreous: No cells; OS: A/C: Deep and quiet; Vitreous: Pigmented cells; rare white cells
	04/7/2011: OD: 0.56; OS: 0.56	04/7/2011: OD: 19.55; OS: 6.32	04/07/2011: MTX 400 mcg/0.1 cc; RTX 1 mg/0.1 cc	04/07/2011: OD: A/C: Rare cells; Vitreous: No cells; OS: A/C: Deep and quiet; vitreous: Pigmented cells; rare white cells
Case 9			01/18/2010: OD: MTX 400 mcg in 0.08 cc; RTX 1 mg/0.08 cc	
			01/20/2010: OS: MTX 400 mcg/0.08 cc; RTX 1 mg/0.08 cc	
			01/17/2011: OU: MTX 200 mcg/0.1 cc; RTX 1 mg/0.1 cc	
				02/09/2011: OD: A/C: Trace to 1+ cells; Vitreous: Post vitreous detachment, 3+ vitreous cells; OS: A/C: Trace cells; Vitreous: Post vitreous detachment, 1+ vitreous debris
	02/11/2011: OD: 21.75	02/11/2011: OD: 1215	02/11/2011: OD: MTX 400 mcg/0.1 cc; RTX 1 mg/0.1 cc	
	02/23/2011: OD: 1.48; OS: 2.04	02/23/2011: OD: 102.28; OS: 136.78	02/23/2011: OU: MTX 400/0.1 cc; RTX 1 mg/0.1 cc	02/23/2011: Injection only; No eye exam available
			02/28/2011: OS: Ceftazidine 2.25 mg/0.1 cc; Vancomycin 1000 mcg/0.1 cc	
	03/23/2011: OD: 1.59; OS: 0.56	03/23/2011: OD: 33.99; OS: 25.36	03/23/2011: OU: MTX 400 mcg/0.1 cc	03/23/2011: OD: A/C: 2+ cell, 1+ flare, 1+ NS; Vitreous: ?; OS: A/C: 1–2+ cell; 1+ NS; Vitreous: 2+ cell
	04/13/2011: OD: 0.56; OS: O.56	04/13/2011: OD: 9.25; OS: 4.91	04/13/2011: OU: MTX 400/0.1 cc	04/13/2011: OD: A/C: 3+ NS; Vitreous: No infiltrates; OS: A/C: 2+ NS; Vitreous: No infiltrates
Case 10	02/24/2011: OD: 97.38; OS: 96.85	02/24/2011: OD: 14.29; OS: 27.12		02/24/2011: No eye exam available
	04/12/2011: OD: 263.9; OS: 151.29	04/12/2011: OD: 24.16; OS: 24.22		04/12/2011: OD: A/C: Deep and quiet; Vitreous: Significant anterior vitreous cells mixed with residual anterior vitreous sheath; OS: A/C: Deep and quiet; Vitreous: Rare vitreous cells present
	05/06/2011: OD: 381.44; OS:–	05/06/2011: OD: 128.3; OS: –	05/06/2011: OD: MTX 400 mcg/0.1 cc; RTX 1 mg/0.1 cc	05/06/2011: OD: A/C: Rare white cells; Vitreous: Anterior hyaloids face densely covered with white cells: OS: A/C: No cells, rare white cells; Vitreous: No cells

*A/C* Anterior chamber, *MTX* Methotrexate, *OD* Oculus dexter, *OS* Oculus sinister, *OU* oculus uterque *RTX* Rituximab.

**Table 3:** Profiles of patients, interleukin concentrations, and dates of intraocular treatment in patients with uveitis (Cases 11–17).

Case #	Sex/Age	Comorbidities	VA _Initial_	VA _Recent_	Diagnosis	Interleukin -10 concentration (pg/ml)	Interleukin-6 concentration (pg/ml)	Treatment
Case 11	74-year-old Caucasian female	Shingles involving the left brow area	OD: 20/100 (NI)	OD: 200 E at 8′	Diagnosed with idiopathic uveitis	12/21/2010: OD: 0.69	12/21/2010: OD: 95.18	None
			OS: 20/60 (NI)	OS: 20/25	PPV at an outside provider (12/2009) demonstrated CD10-positive B cell population			
					ACE, CBC, CXR, and lysozyme were within normal limits; the patient was HLA-A29 negative; β2 microglobulin was elevated			
					Vitreous cytology negative for malignancy from PPV (01/29/2010)			
Case 12	91-year-old Caucasian female	Uterine cancer,Skin cancer (BCC, SCC), Leaky heart valve	OD: 20/60^+2^ (20/50^−2^)	OD: 20/40^−1^ (NI)	Diagnosed with idiopathic uveitis	07/07/2010: OD: <0.56; OS: <0.56	07/07/2010: OD: 232.16; OS: 246.41	None
			OS: 20/150^−1^ (20/100^−1^)	OS: 20/30^+1^ (NI)	QuantiFERON-TB and β2 microglobulin were elevated			
					Vitreous cytology negative for malignancy from PPV (07/07/2010); bacterial and fungal cultures of the vitreous also negative			
					ACE, Lyme titers, and syphilis IgG and IgM within normal limits			
Case 13	81-year-old Caucasian male	Type II diabetes mellitus, chronic obstructive pulmonary disease, bladder cancer, and history of deep vein thrombosis	OD: 20/30 (NI)	OD: 20/25	Diagnosed with chronic granulomatous panuveitis, likely secondary to fungal infection			06/02/2010 OS: Triamcinolone
			OS : 200 E at 10′ (NI)	OS: 20/40	Presented to an outside provider with ocular symptoms; complete uveitis workup was negative except for an elevated ESR of 66			07/09/2010 OS: Triamcinolone
					CXR was significant only for hiatal hernia and changes consistent with chronic obstructive pulmonary disease	07/22/2010: OS: 2.87	07/22/2010: OS: 936.24	
					Blood and fungal cultures were negative with no evidence of abscess on CT of chest/abdomen/pelvis.			
					Vitreous pathology negative for malignancy, but notable for marked acute inflammatory response with focal granulomas from PPV (08/18/2010)			
					AFB smear, Gram stain, and GMS stain were negative. However, retinal sampling was significant for one cluster of *Candida albicans*			
Case 14	73-year-old Caucasian male	Papillary thyroid carcinoma, Sarcoidosis, Type II diabetes mellitus, hypercalcemia, vitamin D deficiency, hypertension, and chronic renal insufficiency	OD: 20/150 (20/100^+1^)	OD: 20/150 (NI)	Diagnosed with uveitis likely secondary to Sarcoidosis	08/2010: OS: 4.84	08/2010: OS: 805.91	None
			OS: 20/400 (20/200)	OS: 20/150 (NI)	Vitreous pathology negative for malignancy but notable for a mixed inflammatory infiltrate consistent with a reactive process from PPV (08/27/10)			
Case 15	56-year-old Caucasian male	Sarcoidosis	OD: 20/60^−2^ (NI)	OD: 20/70^+2^ (NI)	Diagnosed with uveitis likely secondary to Sarcoidosis.			2/17/2011: OD: MTX 400 mcg/0.1 cc
			OS: 20/25^−2^	OS: 20/25^−2^	CT of chest positive for enlarged mediastinal and hilar lymph nodes, and levels of ACE were elevated	04/28/2011: OD: <0.56	04/28/2011: OD: 72.73	04/28/2011: OD: MTX 400 mcg/0.1 cc
						05/06/2011OS: <0.56	05/06/2011OS: 16.52	
Case 16	59-year-old African American male	Sarcoidosis with uveitis diagnosed in 08/2005	OD: 200 E at 9′	OD: 20/40 (20/40^+2^)	Diagnosed with uveitis likely secondary to Sarcoidosis			08/2006: OD: Triamcinolone
			OS: 20/15^−1^	OS: 20/20	Vitreous pathology negative for malignancy and showed only a mixed inflammatory infiltrate from PPV (10/2006)			08/2006: OD: Periocular steroid injection
								09/2007:OD: Dexamethasone and MTX; 2 mg subconjunctival Dexamethasone
						03/28/2011: OD: <0.56	03/28/2011: OD: 28.33	03/28/11: OD: Bevacizumab 1.25 mg/0.05 cc, MTX 400 mcg/0.1 cc
Case 17	12-year-old Caucasian female	None	OD: 20/150^+1^ (NI)	OD: 20/125	Diagnosed with idiopathic intermediate uveitis	05/16/2011: OD: <0.56	05/16/2011: OD: 114.87	05/16/2011: OD: MTX 400 mcg/0.1 cc
			OS: 20/150^+2^ (20/60^+2^)	OS: 20/150 (20/100-1)				

*ACE* Angiotensin-converting enzyme, *AFB* Acid-fast bacillus, *BCC* Basal cell carcinoma, *CBC* Complete blood count, *CT* Computed tomography, *CXR* Chest x-ray, *ESR* Erythrocyte sedimentation rate, *GMS* Grocott’s methenamine silver, *HLA* Human leukocyte antigen, *OD* Oculus dexter, *OS* Oculus sinister, *OU* Oculus uterque, *PPV* Pars plana vitrectomy, *SCC* Squamous cell carcinoma, *TB* Tuberculosis, *VA* Visual acuity.

The threshold of sensitivity for the IL-10 and Il-6 assays was 1.0 pg/mL and 4.88 pg/mL, respectively. Interleukin-10 levels <0.56 pg/mL and IL-6 levels <1.67 pg/mL represented concentrations below the lower boundary of detectable for our assay; therefore, these values were used in our analysis to represent undetectable levels for the respective cytokine. Selecting the highest IL-10 and lowest IL-6 level for each patient with lymphoma and the lowest IL-10 and highest IL-6 level for each patient with uveitis yielded a total of 10 data points from 10 patients with lymphoma and 7 data points from 7 patients with uveitis. Interleukin levels were tabulated for each group and are reported in Table 4 along with mean, median, and range values.

**Table 4:** Interleukin concentrations in patients with lymphoma and uveitis.

	[IL-10] (pg/ml)		[IL-6] (pg/ml)		IL-10/IL-6	
	Lymphoma	Uveitis	Lymphoma	Uveitis	Lymphoma	Uveitis
1	0.56	0.56	1.67	28.33	0.002644	0.002272
2	1.73	0.56	1.67	72.73	0.0346	0.003065
3	9.21	0.56	4.91	95.18	0.12104	0.004875
4	9.59	0.56	6.9	114.87	0.7484	0.006006
5	21.75	0.69	9.79	246.41	0.9408	0.007249
6	33.74	2.87	14.29	805.91	1.0359	0.0077
7	34.45	4.84	46.03	936.24	4.4297	0.019767
8	72.19		211.79		22.088	
9	152.41		277.38		26.693	
10	381.44		278.75		43.228	
	Mean = 71.71 pg/mL	Mean = 1.52 pg/mL	Mean = 85.32 pg/mL	Mean = 328.52	Mean = 9.93	Mean = 7.28e-3
	Median = 27.75 pg/mL	Median = 0.56 pg/mL	Median = 12.04 pg/mL	Median = 114.87	Median = 0.988	Median = 6.01e-3
	Range = 0,56–381.44 pg/mL	Range = 0.56–4.84 pg/mL	Range = 1.67–278.75 pg/mL	Range = 28.33–936.24	Range = 0.002644–43.23	Range = 0.002272–0.019767

### Statistical Analysis

**Figure 1:**
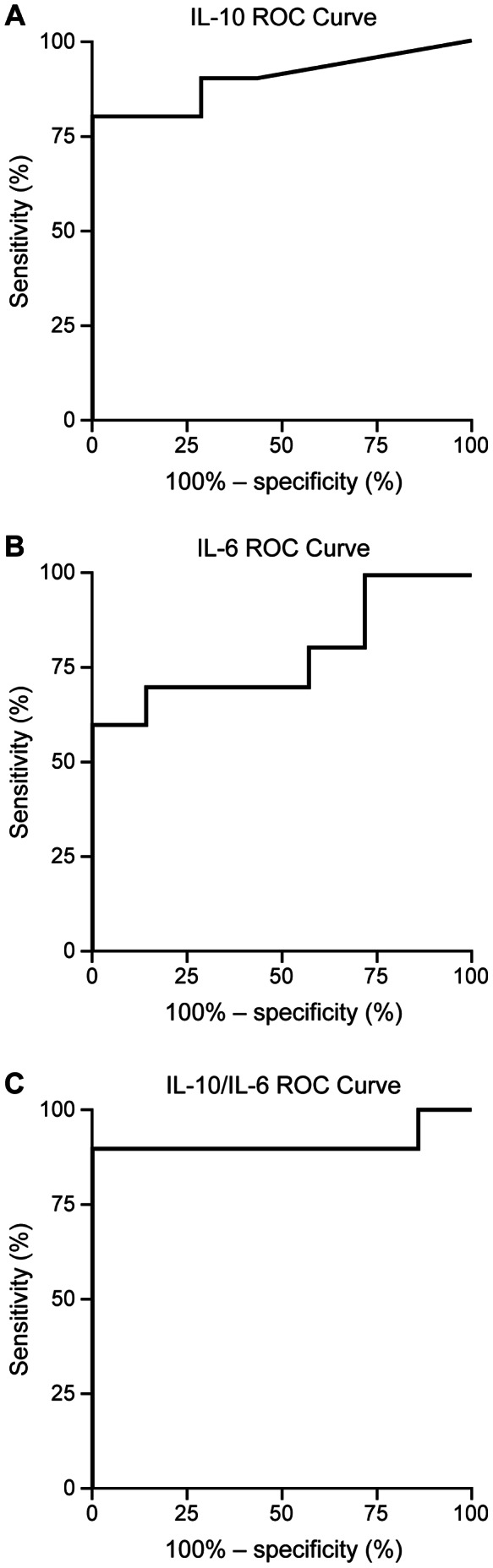
Empirical ROC curve using IL threshold values. a) IL-10 ROC curve, b) IL-6 ROC curve, c) IL-10/IL-6 ROC curve. The vertical axis represents the true positive rate (sensitivity), and the horizontal axis represents the false positive rate (1-specificity). The curve was generated by dichotomizing IL values into one of two groups (i.e., lymphoma vs. no lymphoma) to determine statistical indices associated with discrete threshold values.

ROC analysis with estimate of areas under the curves was completed to identify threshold values of IL-10 (Fig. 1a), IL-6 (Fig. 1b), and IL-10/IL-6 (Fig. 1c). Area under the ROC curve was 0.900 for IL-10 (95% CI, 0.744–1.0; p = 0.006), 0.786 for IL-6 (95% CI, 0.563–1.0 p = ns), and 0.914 for IL-10/IL-6 (95% CI, 0.751–1.0; p = 0.005). Sensitivity, specificity, and likelihood ratio are reported for discrete threshold values of IL-10 (Table 5), IL-6 (Table 6), and IL-10/IL-6 (Table 7) as derived by GraphPad Prism.

**Table 5:** Statistical indices for threshold values of IL-10 for discriminating lymphoma from uveitis.

Cutoff	Sensitivity%	95% CI	Specificity%	95% CI	Likelihood ratio
>0.6250	90.00	55.50% to 99.75%	57.14	18.41% to 90.10%	2.100
>1.210	90.00	55.50% to 99.75%	71.43	29.04% to 96.33%	3.150
>2.300	80.00	44.39% to 97.48%	71.43	29.04% to 96.33%	2.800
>3.855	80.00	44.39% to 97.48%	85.71	42.13% to 99.64%	5.600
>7.025	80.00	44.39% to 97.48%	100.0	59.04% to 100.0%	
>9.400	70.00	34.75% to 93.33%	100.0	59.04% to 100.0%	
>15.67	60.00	26.24% to 87.84%	100.0	59.04% to 100.0%	
>27.75	50.00	18.71% to 81.29%	100.0	59.04% to 100.0%	
>34.10	40.00	12.16% to 73.76%	100.0	59.04% to 100.0%	
>53.32	30.00	6.674% to 65.25%	100.0	59.04% to 100.0%	
>112.3	20.00	2.521% to 55.61%	100.0	59.04% to 100.0%	
>266.9	10.00	0.2529% to 44.50%	100.0	59.04% to 100.0%	
					

CI Confidence Interval.

**Table 6:** Statistical indices for threshold values of IL-6 for discriminating lymphoma from uveitis.

Cutoff	Sensitivity%	95% CI	Specificity%	95% CI	Likelihood ratio
<3.290	20.00	2.521% to 55.61%	100.0	59.04% to 100.0%	
<5.905	30.00	6.674% to 65.25%	100.0	59.04% to 100.0%	
<8.345	40.00	12.16% to 73.76%	100.0	59.04% to 100.0%	
<12.04	50.00	18.71% to 81.29%	100.0	59.04% to 100.0%	
<21.31	60.00	26.24% to 87.84%	100.0	59.04% to 100.0%	
<37.18	60.00	26.24% to 87.84%	85.71	42.13% to 99.64%	4.200
<59.38	70.00	34.75% to 93.33%	85.71	42.13% to 99.64%	4.900
<83.96	70.00	34.75% to 93.33%	71.43	29.04% to 96.33%	2.450
<105.0	70.00	34.75% to 93.33%	57.14	18.41% to 90.10%	1.633
<163.3	70.00	34.75% to 93.33%	42.86	9.899% to 81.59%	1.225
<229.1	80.00	44.39% to 97.48%	42.86	9.899% to 81.59%	1.400
<261.9	80.00	44.39% to 97.48%	28.57	3.669% to 70.96%	1.120
<278.1	90.00	55.50% to 99.75%	28.57	3.669% to 70.96%	1.260
<542.3	100.0	69.15% to 100.0%	28.57	3.669% to 70.96%	1.400
<871.1	100.0	69.15% to 100.0%	14.29	0.3610% to 57.87%	1.167

CI Confidence Interval.

**Table 7:** Statistical indices for threshold values of IL-10/IL-6 for discriminating lymphoma from uveitis.

Cutoff	Sensitivity%	95% CI	Specificity%	95% CI	Likelihood ratio
>0.002458	100.0	69.15% to 100.0%	14.29	0.3610% to 57.87%	1.167
>0.002855	90.00	55.50% to 99.75%	14.29	0.3610% to 57.87%	1.050
>0.00397	90.00	55.50% to 99.75%	28.57	3.669% to 70.96%	1.260
>0.005440	90.00	55.50% to 99.75%	42.86	9.899% to 81.59%	1.575
>0.006627	90.00	55.50% to 99.75%	57.14	18.41% to 90.10%	2.100
>0.007474	90.00	55.50% to 99.75%	71.43	29.04% to 96.33%	3.150
>0.01373	90.00	55.50% to 99.75%	85.71	42.13% to 99.64%	6.300
>0.02718	90.00	55.50% to 99.75%	100.0	59.04% to 100.0%	
>0.07782	80.00	44.39% to 97.48%	100.0	59.04% to 100.0%	
>0.4347	70.00	34.75% to 93.33%	100.0	59.04% to 100.0%	
>0.8446	60.00	26.24% to 87.84%	100.0	59.04% to 100.0%	
>0.9884	50.00	18.71% to 81.29%	100.0	59.04% to 100.0%	
>2.733	40.00	12.16% to 73.76%	100.0	59.04% to 100.0%	
>13.26	30.00	6.674% to 65.25%	100.0	59.04% to 100.0%	
>24.39	20.00	2.521% to 55.61%	100.0	59.04% to 100.0%	
>34.96	10.00	0.2529% to 44.50%	100.0	59.04% to 100.0%	

CI Confidence Interval.

**Figure 2:**
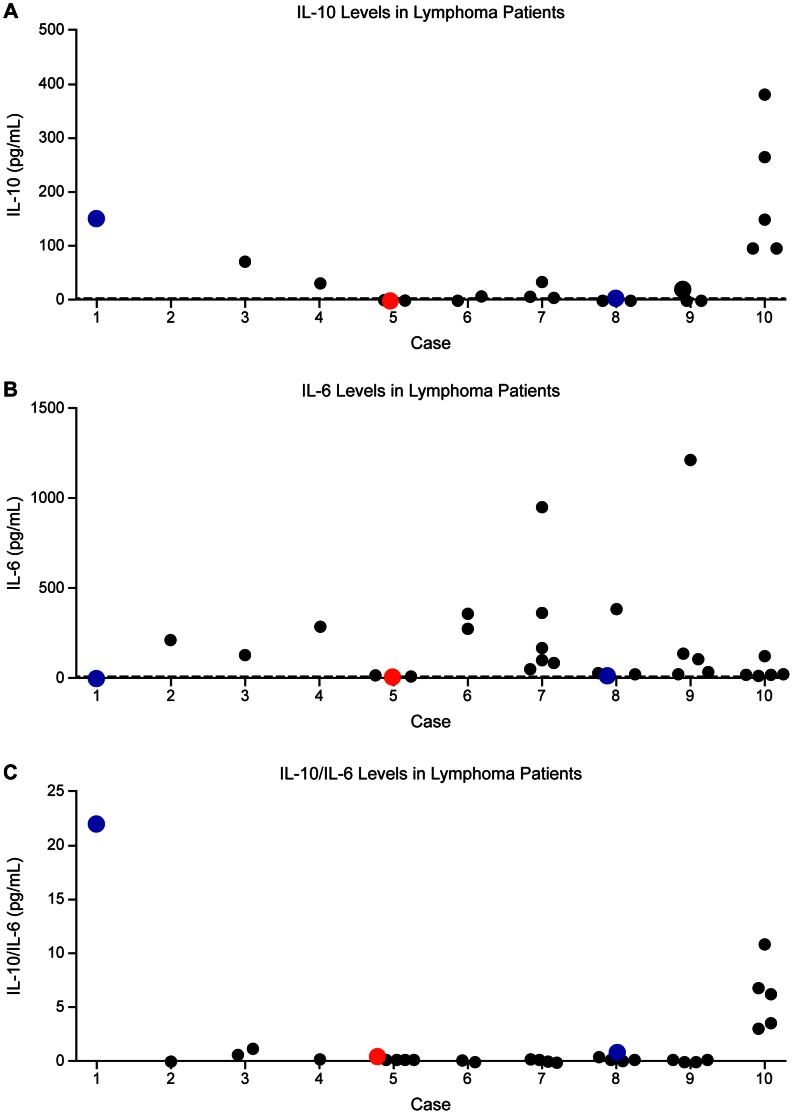
Distribution of IL values in patients with vitreoretinal lymphoma. a) IL-10, b) IL-6, c) IL-10/IL-6. The vertical axis represents IL concentration or, in the case of IL-10/IL-6, the IL-10/IL-6 ratio. Black dots represent data points from individual dates of paracentesis. Red dots represent those IL values during the month of diagnosis of ocular disease whereas blue dots represent those IL values at the time of disease relapse. The dashed line represents the assay’s threshold of sensitivity, which for IL-10 is 1.0 pg/mL and for IL-6 is 4.88 pg/mL.

**Figure 3:**
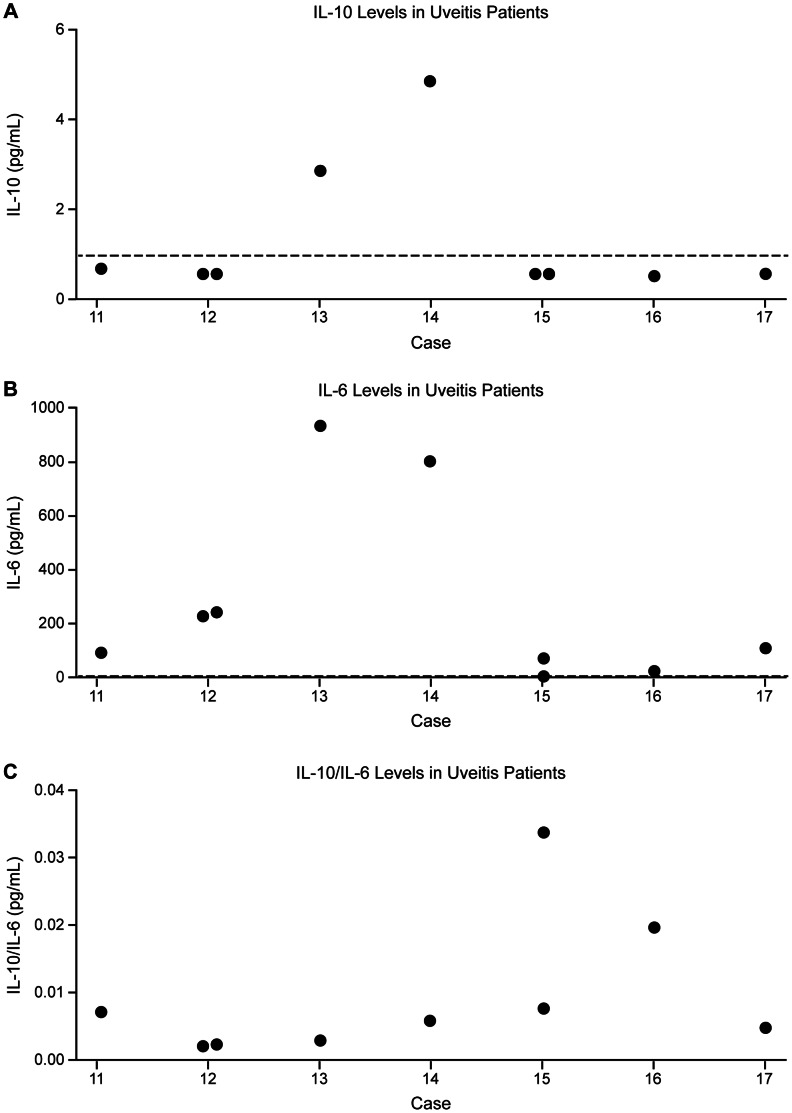
Distribution of IL values in patients with uveitis. a) IL-10, b) IL-6, c) IL-10/IL-6. The vertical axis represents IL concentration or, in the case of IL-10/IL-6, the IL-10/IL-6 ratio. Black dots represent data points from individual dates of paracentesis. The dashed line represents the assay’s threshold of sensitivity, which for IL-10 is 1.0 pg/ml and for IL-6 is 4.88 pg/mL.

Additionally, we analyzed each cytokine marker to see if a significant difference in the distribution of IL values exists between eyes with lymphoma and uveitis. In a rank-sum test, IL-10 values were significantly higher in eyes with lymphoma than uveitis (U = 7.0; two-tailed p = 0.004). Similarly, IL-10/IL-6 levels were significantly elevated in eyes with lymphoma (U = 6.0; two-tailed p = 0.003). Conversely, there was no significant difference in IL-6 levels between the lymphoma and uveitis groups (U = 15.0; two-tailed p = ns). The distribution of all available IL-10, IL-6, and IL-10/IL-6 values for each patient with lymphoma, including multiple data points from the same eye, is graphically depicted in Fig. 2a, Fig. 2b, and Fig. 2c, respectively. Similarly, the distribution of all available IL-10, IL-6, and IL-10/IL-6 levels in uveitis patients is represented in Fig. 3a, Fig. 3b, and Fig. 3c, respectively. However, the Mann-Whitney calculations above were based on the selected data points as outline in the Materials and Methods section.

### Effect of Intravitreal Methotrexate (MTX) and Rituximab (RTX) on IL-10 Levels

Interleukin-10, IL-6, and IL-10/IL-6 response to treatment with intraocular MTX and RTX are reported in three patients. In two cases (Cases 8 & 9), serial intraocular treatment was associated with reduction in aqueous IL-10 levels over time. In a third case (Case 10), IL-10 levels increased steadily in the absence of treatment.

### Case Examples

#### Case 8

**Figure 4:**
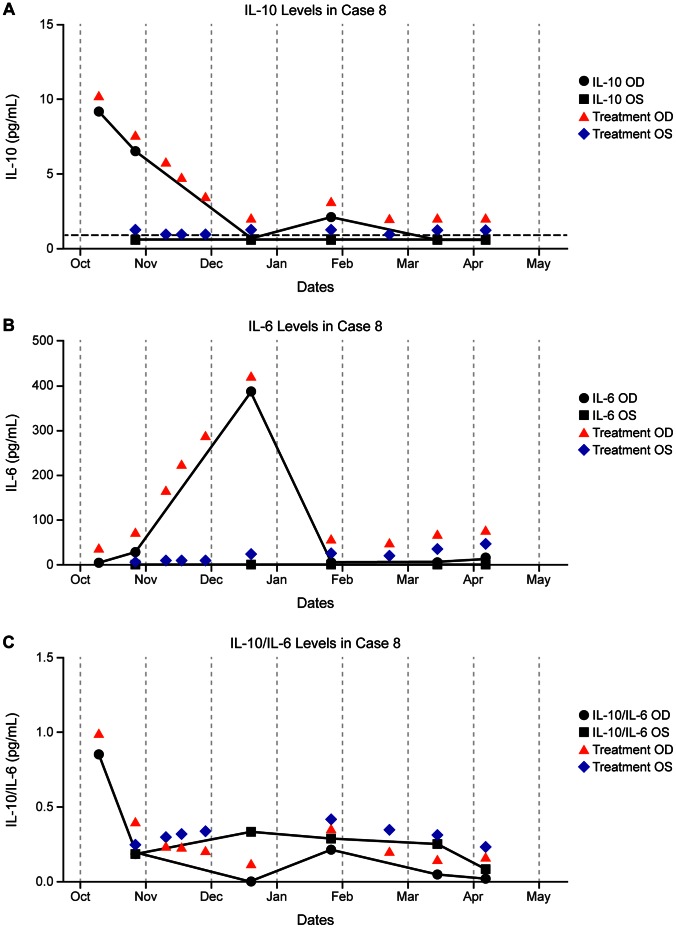
IL concentrations and timing of intraocular treatment in a patient with unilateral lymphoma (Case 8). a) IL-10, b) IL-6, c) IL-10/IL-6. The vertical axis represents IL levels. Red arrows indicate intraocular treatment in the right eye, and blue diamonds indicate intraocular treatment in the left eye.

A 54-year-old man with systemic lymphoma underwent induction chemotherapy with high-dose MTX and six cycles of R-CHOP chemotherapy, followed by consolidation with BEAM conditioning and autologous stem cell transplant (SCT) in March 2009 to complete remission. In October 2008, he was diagnosed with vitreoretinal lymphoma and treated with periodic intraocular RTX with good clinical response. The patient developed relapse of ocular lymphoma in October 2010 and was treated with intraocular MTX, RTX, and corticosteroids. The IL-10 level was elevated in the right eye at the time of ocular relapse but declined to undetectable levels by March 2011 while receiving intraocular treatment. Interleukin-10 levels remained undetectable in the fellow eye. The patient has since been in complete remission. Interleukin-10, IL-6, and IL-10/IL-6 response to intravitreal treatment is displayed in graph form (Fig. 4a–c).

#### Case 9

**Figure 5:**
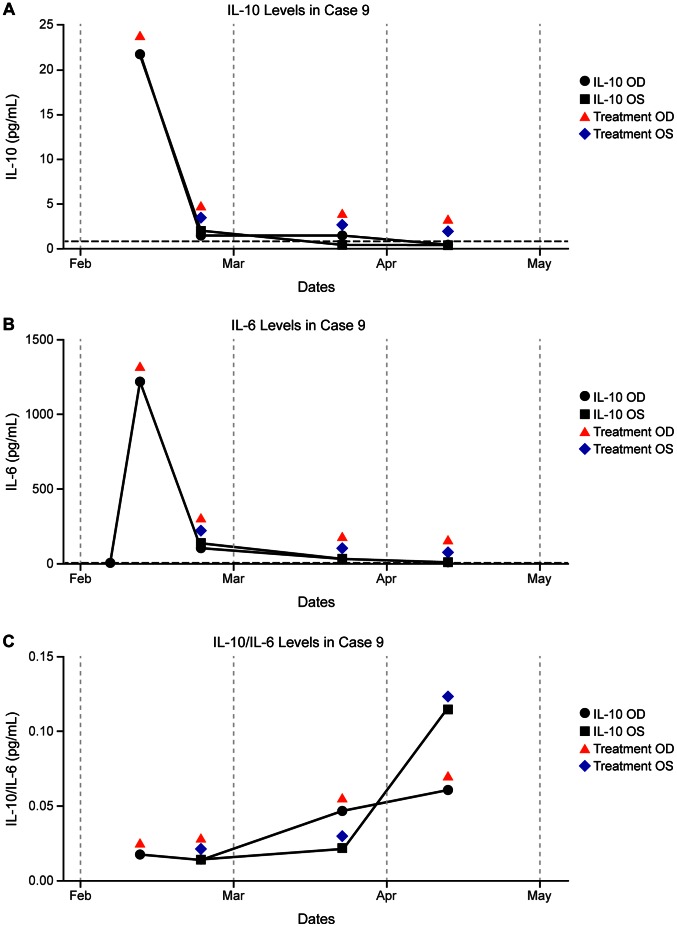
IL concentrations and timing of intraocular treatment in a patient with bilateral lymphoma (Case 9). a) IL-10, b) IL-6, c) IL-10/IL-6. The vertical axis represents IL levels. Red arrows indicate intraocular treatment in the right eye, and blue diamonds indicate intraocular treatment in the left eye.

A 68-year-old woman with history of breast cancer and CNS lymphoma, diagnosed in July 2009, underwent induction chemotherapy with high-dose MTX to complete remission, followed by consolidation with BEAM conditioning and autologous SCT in January 2010. In December 2009, she was seen by an outside provider for ocular symptoms and treated with corticosteroids for presumed optic neuritis. Upon presenting to us in January 2010, the patient was clinically diagnosed with vitreoretinal lymphoma and initiated on maintenance intraocular MTX and RTX for bilateral disease. Pars plana vitrectomy in the right eye in February 2011 confirmed the diagnosis of diffuse large B cell lymphoma. The IL-10 level in the right eye was markedly elevated at the time of PPV and minimally elevated in the left eye later that month. While receiving intraocular treatment, there was a steady decline in IL-10 to undetectable levels by April 2011 in both eyes. Incidentally, after receiving an intraocular injection in February, the patient developed a self-resolving post-injection sterile endophthalmitis in the left eye. Interleukin-10, IL-6, and IL-10/IL-6 response to intravitreal treatment is displayed in graph form (Fig. 5a–c).

#### Case 10

**Figure 6:**
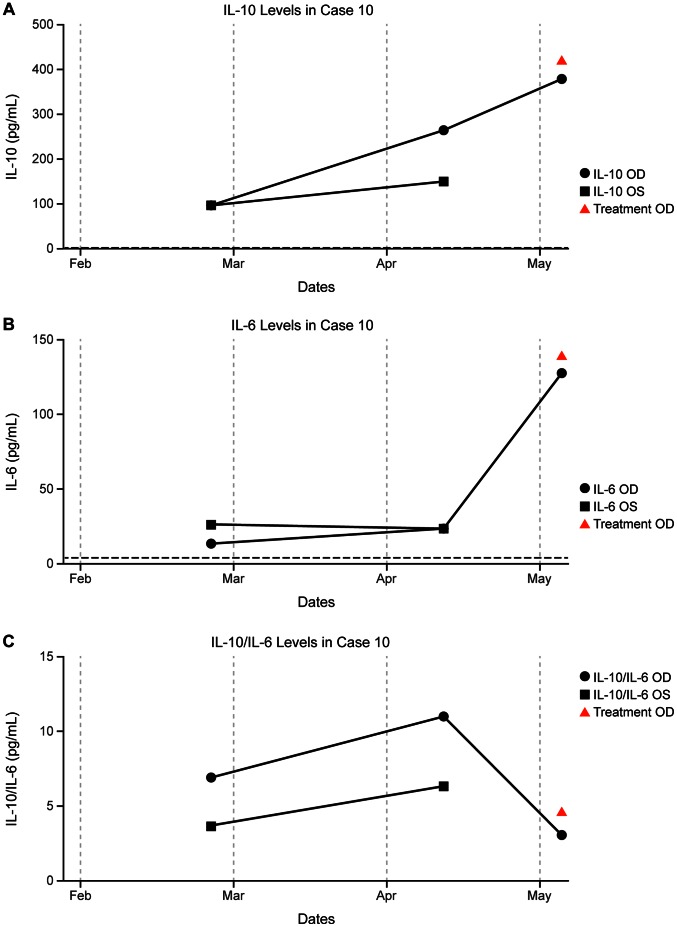
IL concentrations and timing of intraocular treatment in a patient with bilateral lymphoma (Case 10). a) IL-10, b) IL-6, c) IL-10/IL-6. The vertical axis represents IL levels. Red arrows indicate intraocular treatment in the right eye, and blue diamonds indicate intraocular treatment in the left eye.

A 72-year-old woman, diagnosed with bilateral PVRL by PPV at an outside provider in September 2010, did not receive intraocular treatment prior to her first visit to our clinic in December 2010. Between February and May 2011, IL-10 levels increased markedly in both eyes. In May, the patient again presented to our clinic with worsening vision. Pars plana vitrectomy at that time confirmed PVRL, and intraocular MTX and RTX were administered intraoperatively. Also at this time, the patient was diagnosed with new CNS disease and subsequently underwent six cycles of systemic temozolomide, MTX and RTX with complete response of the CNS lymphoma. The patient underwent PPV in September 2011, followed by treatment with intraocular MTX and RTX in both eyes at her outside provider in November 2011. The patient developed active ocular disease in December 2011 but did not receive intraocular treatment by her provider due to intraocular pressure spike following injections. Interleukin-10, IL-6, and IL-10/IL-6 response to intravitreal treatment is displayed in graph form (Fig. 6a–c).

## Discussion

Intravitreal lymphoma represents a vision and potentially life-threatening ophthalmic condition. Early diagnosis and treatment is, therefore, important. The current gold standard for diagnosis of lymphoma is pars plana vitrectomy with retinal or choroidal biopsy. However, more recent studies have focused on identifying less invasive methods (e.g., intraocular cytokines) of diagnosing lymphoma and monitoring therapeutic response. The goal of our study was to identify *optimal* diagnostic sensitivities and specificities for specific cytokines IL-10, IL-6, and IL-10/IL-6. Prior studies, have demonstrated elevated IL-10 levels in patients with lymphoma [5], [9], [10], [11] as well as elevated IL-10/IL-6 levels in lymphoma compared to uveitis. [9], [10], [11].

Our findings demonstrate that aqueous levels of IL-10 and IL-10/IL-6 are elevated in lymphoma compared to uveitis. Conversely, IL-6 levels did not demonstrate any statistical difference between those patients with uveitis and those with lymphoma, although they tended to be higher in eyes with uveitis. Additionally, in our study, both IL-10 and IL-10/IL-6 cytokine markers were moderately accurate tools for discriminating lymphoma from uveitis. Indeed, when optimal threshold values are utilized, combined sensitivity ≥80% specificity = 100% are achievable for both cytokines. Importantly, in the cases where follow-up was available, aqueous levels of IL-10 and, to a lesser extent, IL-10/IL-6 correlated well with disease activity and treatment response.

In our analysis, we selected the highest IL-10 and lowest IL-6 values in each patient with lymphoma and the lowest IL-10 and highest IL-6 values in each patient with uveitis. Multiple data points from the same eye were, in this way, excluded. Potential threshold values for each of the cytokine markers were then generated using ROC curves. Although this method would naturally overestimate sensitivity and specificity for our cytokines, given the relatively small sample size of our study, this method allowed us to identify the best diagnostic sensitivity and specificity associated with threshold values in our assay for these given markers. This was also the preferred method since patients had anterior chamber sampling done at various times throughout their care and not all patients had aqueous measurements taken at the time of diagnosis.

Unfortunately, many patients undergo a protracted workup period before obtaining an accurate diagnosis. One reason for this is that signs and symptoms of lymphoma can be quite nonspecific; also, patients can transiently respond to intraocular steroids if they are treated under the presumption of having uveitis, further confounding the diagnosis. In light of this, our study did not exclude patients who had already been treated either for lymphoma or uveitis or who had already received a diagnosis prior to being referred to our clinic. Instead, we included all-comers being evaluated for lymphoma within an 18-month period.

As a whole, IL-10 levels were found to parallel clinical findings quite well. However, there was one notable case in which IL levels deviated from clinical exam findings. In Case 10, the patient had an IL-10 concentration = 151.29 pg/mL but was clinically noted to have only rare cells in the vitreous. However, this patient went on to develop recurrent lymphoma months later and likely represented a case of smoldering lymphoma. This demonstrates the difficulty of using only clinical parameters to diagnose lymphoma and the utility of IL levels as an adjunct.

Despite the finding that IL-10 levels were consistently more elevated in lymphoma relative to uveitis, there was sufficient overlap in the range of values, especially at the lower end of the assay, such that a diagnosis of lymphoma likely cannot be excluded on the basis of minimally elevated or even undetectable levels of IL-10. However, elevated IL-10 and IL-10/IL-6 values are indicative of lymphoma. As demonstrated by Case 10, elevated levels of IL-10, even in an otherwise clinically quiescent eye, may suggest impending disease recurrence; clinical disease severity should be monitored closely.

Our study was limited by small sample sizes in all groups. Therefore, further studies are warranted, such as screening interleukin levels at the time of initial presentation; however, this study adds further credence to the accumulating evidence of the utility of aqueous IL-10 levels as a diagnostic adjunct in evaluating lymphoma. To make the diagnosis by the use of only the IL-10 level is most likely not possible at the present time with the assay as we use it. However, it can serve as a useful adjunct to conventional methods of diagnosis and, as shown, for monitoring disease progression and response to treatment. The use of a combination of the cytokine profiles IL-10/IL-6 and IL-10/IFN-gamma was recently shown to be quite effective as a diagnostic adjunct for discriminating lymphoma from uveitis. [13] However, although IL-10/IL-6 was found to be slightly higher in patients with lymphoma, this was not consistently the case in all patients, again suggesting an important role of cytokines as an adjunct for diagnosing lymphoma.

Intraocular MTX and RTX are highly efficacious in the treatment of lymphoma. The latter drug directly targets the B-cell lineage [4] and minimizes side effects associated with MTX. [14], [15] Few studies have monitored the response in interleukin levels to intraocular injections of MTX and/or Rituximab. [16], [17] In one study, which measured IL-10 and IL-6 levels in undiluted aqueous humor before and after completion of intraocular treatment with MTX (weekly 400 µg/50 mL for 6 weeks), the IL-10/IL-6 ratio decreased over the span of the treatment. [16] A similar drop in IL-10/IL-6 after treatment with intravitreal MTX was generally shown in a recent retrospective study reporting MTX resistance in a patient with lymphoma undergoing regular injections with the drug. [18] A more recent study demonstrated the effect of regular intravitreal injections with MTX on aqueous levels of IL-10. [19] This study showed that undetectable levels could be obtained with regular injections and that spikes in IL-10 could correlate with disease recurrence. Our findings help to confirm the usefulness of following IL-10 levels in patients being treated for vitreoretinal lymphoma. Considering that the present technique of determining the amount of vitreous cells present is not very quantitative, this would be a useful adjunct. Though IL-10 levels can be elevated in uveitis, the adjunctive use in following treatment effects in patients with vitreoretinal lymphoma makes the determination of the levels important. [20] This finding suggests that intraocular cytokine analysis may be valuable not only for diagnosis but also for monitoring therapeutic response.

We have, herein, described two cases of lymphoma in which treatment with intraocular MTX and RTX was shown to decrease IL-10 concentrations to undetectable levels over time. Interleukin-6 and IL-10/IL-6 levels were less reliable indicators of response to therapy. Both patients had already completed systemic therapy when IL-10 levels were measured; therefore, any confounding effect by systemic therapy is unlikely. In another patient who did not receive intraocular therapy, IL-10 levels were markedly elevated and continued to increase over time. We have additionally indicated that IL-10 levels appear to correlate quite well with severity of vitreous cell (Table 2 and Table 3). Where there is discrepancy between interleukin levels and the clinical severity of the disease, the clinician should consider all of the available data when considering overall clinical response to therapy. As mentioned previously, elevated IL-10, even in the setting of a quiescent eye, may suggest disease recurrence. When a diagnosis of lymphoma is established, serial measurements of IL-10 is promising as a quantitative tool for monitoring disease status and therapeutic efficacy.

## References

[1] Gunduz K, Pulido JS, McCannel CA, O’Neill BP (2006) Ocular manifestations and treatment of central nervous system lymphomas. Neurosurgical focus 21: E9. 10.3171/foc.2006.21.5.10 17134125

[2] Rajagopal R, Harbour JW (2011) Diagnostic testing and treatment choices in primary vitreoretinal lymphoma. Retina 31: 435–440. 21336066 10.1097/IAE.0b013e31820a6743

[3] Grimm SA, Pulido JS, Jahnke K, Schiff D, Hall AJ, et al (2007) Primary intraocular lymphoma: an International Primary Central Nervous System Lymphoma Collaborative Group Report. Annals of oncology : official journal of the European Society for Medical Oncology/ESMO 18: 1851–1855. 10.1093/annonc/mdm340 17804469

[4] Itty S, Pulido JS (2009) Rituximab for intraocular lymphoma. Retina 29: 129–132. 19202422 10.1097/IAE.0b013e318192f574

[5] Cassoux N, Merle-Beral H, Leblond V, Bodaghi B, Milea D, et al (2000) Ocular and central nervous system lymphoma: clinical features and diagnosis. Ocular immunology and inflammation 8: 243–250. 11262654 10.1076/ocii.8.4.243.6463

[6] Chan CC, Whitcup SM, Solomon D, Nussenblatt RB (1995) Interleukin-10 in the vitreous of patients with primary intraocular lymphoma. American journal of ophthalmology 120: 671–673. 7485372 10.1016/s0002-9394(14)72217-2

[7] Rousset F, Garcia E, Defrance T, Peronne C, Vezzio N, et al (1992) Interleukin 10 is a potent growth and differentiation factor for activated human B lymphocytes. Proceedings of the National Academy of Sciences of the United States of America 89: 1890–1893. 1371884 10.1073/pnas.89.5.1890 PMC48559

[8] Salazar-Onfray F (1999) Interleukin-10: a cytokine used by tumors to escape immunosurveillance. Medical oncology 16: 86–94. 10456656 10.1007/BF02785841

[9] Whitcup SM, Stark-Vancs V, Wittes RE, Solomon D, Podgor MJ, et al (1997) Association of interleukin 10 in the vitreous and cerebrospinal fluid and primary central nervous system lymphoma. Archives of ophthalmology 115: 1157–1160. 9298057 10.1001/archopht.1997.01100160327010

[10] Sugita S, Takase H, Sugamoto Y, Arai A, Miura O, et al (2009) Diagnosis of intraocular lymphoma by polymerase chain reaction analysis and cytokine profiling of the vitreous fluid. Japanese journal of ophthalmology 53: 209–214. 19484437 10.1007/s10384-009-0662-y

[11] Cassoux N, Giron A, Bodaghi B, Tran TH, Baudet S, et al (2007) IL-10 measurement in aqueous humor for screening patients with suspicion of primary intraocular lymphoma. Investigative ophthalmology & visual science 48: 3253–3259. 17591896 10.1167/iovs.06-0031 PMC2078609

[12] GraphPad Prism version 6.0 for Windows, GraphPad Software, La Jolla California USA. Available: www.graphpad.com. Accessed 2013 Jan 13.

[13] Fisson S, Ouakrim H, Touitou V, Baudet S, Ben Abdelwahed R, et al (2013) Cytokine profile in human eyes: contribution of a new cytokine combination for differential diagnosis between intraocular lymphoma or uveitis. PloS one 8: e52385. 23405064 10.1371/journal.pone.0052385 PMC3566156

[14] Smith JR, Rosenbaum JT, Wilson DJ, Doolittle ND, Siegal T, et al (2002) Role of intravitreal methotrexate in the management of primary central nervous system lymphoma with ocular involvement. Ophthalmology 109: 1709–1716. 12208721 10.1016/s0161-6420(02)01125-9

[15] Ohguro N, Hashida N, Tano Y (2008) Effect of intravitreous rituximab injections in patients with recurrent ocular lesions associated with central nervous system lymphoma. Archives of ophthalmology 126: 1002–1003. 18625954 10.1001/archopht.126.7.1002

[16] Sou R, Ohguro N, Maeda T, Saishin Y, Tano Y (2008) Treatment of primary intraocular lymphoma with intravitreal methotrexate. Japanese journal of ophthalmology 52: 167–174. 18661266 10.1007/s10384-008-0519-9

[17] Kawamura H, Yasuda N, Kakinoki M, Sawada T, Sawada O, et al (2009) Interleukin-10 and interleukin-6 in aqueous humor during treatment of vitreoretinal lymphoma with intravitreally injected methotrexate. Ophthalmic research 42: 172–174. 19648778 10.1159/000230879

[18] Sen HN, Chan CC, Byrnes G, Fariss RN, Nussenblatt RB, et al (2008) Intravitreal methotrexate resistance in a patient with primary intraocular lymphoma. Ocular immunology and inflammation 16: 29–33. 18379939 10.1080/09273940801899764 PMC2561282

[19] Saleh M, Nikolitch K, Bourcier T, Speeg C, Gaucher D (2012) Repeated IL-10 measurement in aqueous humor and OCT imaging are valuable tools to monitor intraocular lymphoma treated with intravitreal injections of methotrexate. Graefe’s archive for clinical and experimental ophthalmology = Albrecht von Graefes Archiv fur klinische und experimentelle Ophthalmologie 250: 761–764. 10.1007/s00417-011-1718-5 21755434

[20] van Kooij B, Rothova A, Rijkers GT, de Groot-Mijnes JD (2006) Distinct cytokine and chemokine profiles in the aqueous of patients with uveitis and cystoid macular edema. American journal of ophthalmology 142: 192–194. 16815285 10.1016/j.ajo.2006.02.052

